# Treatment Efficacy of Theophylline in ADCY5‐Related Dyskinesia: A Retrospective Case Series Study

**DOI:** 10.1002/mds.30170

**Published:** 2025-03-13

**Authors:** Dirk Taenzler, Frank Hause, Andreas Merkenschlager, Andrea Sinz

**Affiliations:** ^1^ Department of Pharmaceutical Chemistry and Bioanalytics Martin Luther University Halle‐Wittenberg Halle (Saale) Germany; ^2^ Center for Structural Mass Spectrometry Martin Luther University Halle‐Wittenberg Halle (Saale) Germany; ^3^ Section for Molecular Cell Biology, Faculty of Medicine Institute of Molecular Medicine, Martin Luther University Halle‐Wittenberg Halle (Saale) Germany; ^4^ Department of Pediatrics University of Leipzig Medical Center Leipzig Germany

**Keywords:** ADCY5, dyskinesia, genetic disorder, theophylline

## Abstract

**Background:**

ADCY5‐related dyskinesia is a rare disorder caused by mutations in the ADCY5 gene resulting in abnormal involuntary movements. Currently, there are no standardized guidelines to treat this condition.

**Objective:**

The aim of this study was to evaluate the efficacy of theophylline administration in improving symptoms and quality of life in patients with ADCY5‐related dyskinesia.

**Methods:**

A retrospective study was conducted involving 12 patients (aged 2–41 years) with ADCY5‐related dyskinesia. Participants completed a questionnaire about theophylline administration, including dosage, improvement of symptoms, adverse effects, and changes in quality of life. Data were analyzed for reported efficacy and side effects.

**Results:**

Theophylline administration demonstrated substantial efficacy, with 92% (11 of 12) of patients reporting significant improvements in their movement disorders. The average improvement score was 7.0 ± 1.9 (mean ± SD) on a 10‐point scale. Notable improvements included reductions in severity and frequency of episodes, improved gait, more independent mobility, psychosocial well‐being, and quality of sleep. Adverse effects were reported by 6 patients, including dystonia, worsening of speech, headaches, nausea, impaired sleep, and agitation.

**Conclusions:**

Theophylline shows substantial promise as a treatment option for ADCY5‐related dyskinesia, improving various aspects of patients' quality of life and movement disorder symptoms. Further research is needed to optimize dosing, to understand long‐term effects, and to explore combinational drug therapies. Despite the small cohort size and the retrospective nature of this study, the results support theophylline administration to decrease dyskinetic movements and enhance overall quality of life in patients. © 2025 The Author(s). *Movement Disorders* published by Wiley Periodicals LLC on behalf of International Parkinson and Movement Disorder Society.

ADCY5‐related dyskinesia is a rare neurological condition caused by mutations in the ADCY5 gene that codes for the enzyme adenylyl cyclase 5. This enzyme is essential for intracellular signaling, as it converts ATP into cyclic AMP (cAMP), a secondary messenger involved in numerous cellular functions. Mutations in the ADCY5 gene cause increased enzymatic activity,^1^ leading to an overproduction of cAMP and clinically resulting in abnormal involuntary movements referred to as dyskinesia.^2^

ADCY5‐related dyskinesia manifests through a variety of impaired voluntary movements, including, but not limited to, choreoathetosis, dystonia, myoclonus, and/or axial hypotonia,^3^ ultimately resulting in poor motor control. These symptoms typically emerge in infancy or early childhood, affecting the limbs, face, and neck.^4^ The movements are often continuous during waking hours and can disrupt sleep. They tend to worsen with stress, fatigue, or illness but may also occur without any identifiable trigger.^4^ The majority of individuals diagnosed with ADCY5‐related dyskinesia are simplex cases, meaning they are the only affected member in their family, and their condition arises from a de novo pathogenic variant. Diagnosis is primarily based on clinical presentation and confirmed through genetic testing to identify mutations in the ADCY5 gene.^5^, ^6^, ^7^

Based on case reports, patients with ADCY5‐related dyskinesia may benefit from deep brain stimulation (DBS).^8^, ^9^, ^10^ Bilateral pallidal DBS can significantly reduce hyperkinetic movements and paroxysmal episodes. While there is generally partial improvement, patients often remain impaired and daily living activities may not significantly improve.^11^

Currently, there are no randomized controlled trials or consensus guidelines for treating ADCY5‐related dyskinesia. Case reports suggest that benzodiazepines, such as clonazepam, clobazam, diazepam, and lorazepam, can help in managing dyskinetic episodes, and also those associated with sleep.^8^ In a retrospective study involving 30 individuals, a beneficial effect of caffeine on hyperkinetic movements was observed.^12^ This effect is thought to be attributed to the attenuating action of caffeine as an adenosine A2A antagonist, which is believed to lead to a reduction of cAMP levels in the striato‐pallidal projection neurons.^12^ The purpose of the present study was to prove a comparable mechanism of action for caffeine and theophylline. As theophylline exhibits a higher affinity for the adenosine A2A receptor, its efficacy was assumed to be higher than that of caffeine for treating ADCY5‐related dyskinesia.^13^

We were able to demonstrate that treatment with the purine derivatives caffeine, theophylline, and istradefylline reduced ADCY5‐catalyzed cAMP production in cell lines overexpressing wild‐type and mutant ADCY5. The most pronounced effects on cAMP reduction were observed in ADCY5 R418W mutant cells,^13^ a mutation found in the majority of individuals with ADCY5‐related dyskinesia.^14^ Subsequent to these findings, a slow‐release theophylline formulation was administered to a preschool‐aged patient with ADCY5‐related dyskinesia, resulting in a striking improvement of symptoms, surpassing the effects of caffeine that had previously been administered to the same patient.^13^

Based on these encouraging results, we here systematically present the first comprehensive account of the effects of theophylline administration in a series of retrospective reports from patients with ADCY5‐related dyskinesia.

## 1 Patients and Methods

Patients and/or their parents, if the patients were too young or unable to complete the questionnaire themselves, were asked to fill out a form that was adapted from a questionnaire developed for a previous study.^12^ The questionnaire targeted individuals who had administered a slow‐release formulation of theophylline to treat ADCY5‐related dyskinesia. The questionnaire was made available to interested participants via the patient organization portal adcy5.org. It was provided in German, English, French, and Spanish. Interested individuals were given the opportunity to answer the questionnaire anonymously. The questionnaire was uploaded by one of the authors (D.T.), who was also responsible for collecting and anonymizing the responses. Anonymized forms were subsequently analyzed (by A.M.). The type of symptoms were discussed internally within the patient organization group with the input of qualified medical personnel. Also, the administration of other drugs was internally discussed within the patient organization and was overseen by medical personnel.

The aim of the ADCY5 questionnaire for parents was to systematically gather comprehensive information about symptoms, treatment responses, and patients' overall quality of life with ADCY5‐related movement disorders and potential changes following theophylline administration. In addition to demographic questions, the questionnaire included items related to the patients' genotype, cardinal dyskinetic symptoms and their frequency, current theophylline dosage, positive and adverse effects of theophylline administration, as well as former and ongoing (co)medications for the treatment of ADCY5‐related dyskinesia.

The study protocol was approved by the Ethics Committee of the Medical Faculty, University of Leipzig on April 30, 2024, and listed in the German Clinical Trials Register (DRKS00034720) on July 25, 2024. All patients (or parents) gave their written consent to participate in this study. All data produced in the present study are available upon reasonable request to the authors. The questionnaire provided to the patients is available as online Data S1.

## 2 Results

### 2.1 Demographics

The study included 12 patients, comprising 7 females and 5 males. The patients' age ranged from 2 to 41 years, with a median age of 8 years. All patients were characterized by ADCY5‐related dyskinesia with a diverse range of baseline hyperkinetic and paroxysmal movement disorders. Genotyping data of the ADCY5 gene was provided by the 12 participants in the questionnaire and confirmed mutations in the ADCY5 gene, with R418W being the most prominent mutation, present in 9 of 12 individuals.

### 2.2 Theophylline Efficacy

Theophylline administration demonstrated substantial efficacy across the study cohort. Of the 12 patients, 11 reported improvements in their movement disorder symptoms, constituting 92% of the cohort. The average improvement score on a 10‐point scale was approximately 7.0, with a standard deviation of 1.9, indicating a high level of efficacy with minor variability.

These improvements were comprehensive, affecting various aspects of the disorder symptoms. Most patients noted a reduction in the severity and frequency of their episodes (8 of 12), with a substantial number reporting reduced duration per episode (8 of 12) (see Table 1).

**TABLE 1:** Characteristics of the patients and theophylline response

Patient No.	Age (years)	Gender (F/M)	Body weight (kg)	ADCY5 mutant	Theophylline dosage daily (mg)	Theophylline dosage daily (mg/kg/day)	Blood level (mg/l)	Improvement of movement disorder (0: No improvement, 10: Drastic improvement)	Improvement of episode severity (less severe) (Yes/No)	Improvement of episode duration (shorter) (Yes/No)	Improvement of episode frequency (less frequent) (Yes/No)	Change in QoL (0: significantly deteriorated, 6: Significantly improved)	Change in QoS (0: No improvement, 10: Drastic improvement)	Adverse effects
1	2	M	10	R418W	200	20.00	10	6	Yes	Yes	Yes	6	0	No
2	34	F	51	M696K	700	13.73	6.1	8.5	Yes	Yes	Yes	6	10	No
3	11	M	32	R418W	400	12.50	10.3	9	Yes	Yes	Yes	6	9	No
4	4	F	13	R418W	400	30.77	25	8	Yes	Yes	Yes	6	9	No
5	7	F	21	R418W	600	28.57	17.5	9	Yes	Yes	Yes	6	9	No
6	9	F	23	R418W	500	21.74	NA	4	No	No	No	5	0	More dystonia
7	7	F	22	R62L, R412L, R45L	550	25.00	14.3	NA	NA	NA	NA	4	2	Episodes of agitation
8	7	M	21	R418W	200	9.52	2	7.5	Yes	Yes	Yes	5	6.5	Worsening of speech
9	11	F	29	R418W	600	20.69	15.4	8	Yes	Yes	Yes	5	8	No
10	10	M	45	R418Q	750	16.67	11.4	8	Yes	Yes	Yes	6	4	Headache (idiopathic origin)
11	6	F	18	R418W	200	11.11	NA	4	NA	NA	NA	6	3	Nausea
12	41	M	66	R418W	1050	15.91	8	4.5	NA	NA	NA	5	0	Episodes of agitation, impaired sleep

Abbreviations: F, female; M, male; QoL: quality of life; QoS: quality of sleep.

Additional patient‐reported outcomes include improvements in gait, more independent mobility, and increased psychosocial well‐being in terms of confidence and happiness, as well as improved quality of sleep (on a 10‐point scale: *M* = 5.0, SD = 3.8), significantly contributing to the overall improved functionality and quality of life reported by the patients.

### 2.3 Theophylline Dosing and Tolerance

Theophylline was administered as a slow‐release formulation at a daily dose ranging between 200 and 1050 mg, adjusted for the patient's body weight (9.5–28.6 mg/kg/day). The narrow therapeutic window of theophylline can efficiently be met by a careful up‐dosing of the drug and by regularly monitoring relevant blood parameters (see Data S2). Patients with a chronic theophylline overdose might experience nonspecific gastrointestinal symptoms.^15^ One distinct advantage of theophylline is, however, its availability as a slow‐release formulation, which makes it suitable for pediatric use despite its narrow therapeutic window.

The medication was generally well‐tolerated. Adverse effects were reported by 6 patients, including dystonia (n = 1), speech impairment (n = 1), headache (idiopathic origin, n = 1), nausea (n = 1), impaired sleep (n = 1), and agitation (n = 2). One patient reported both agitation and impaired sleep.

### 2.4 Other Treatments

Prior to theophylline administration, 10 patients had been treated with different regimens, including caffeine, levetiracetam, and benzodiazepines, with mixed results. After starting theophylline administration, 8 patients continued to use adjunct therapies, such as zopiclone and duloxetine, which were generally well‐tolerated and enhanced overall treatment efficacy.

## 3 Discussion

The present study indicates that theophylline administration substantially improves the symptoms and quality of life in patients with ADCY5‐related dyskinesia, as evidenced by the high level of reported efficacy and alleviated symptoms.

Theophylline administration demonstrated substantial efficacy, with 92% of patients reporting improvements in their movement disorder symptoms. The average improvement score was 7.0 on a 10‐point scale, with a standard deviation of 1.9, indicating a high overall efficacy. Improvements were noted in various symptom domains, including the severity and frequency of episodes, with 8 of 12 patients reporting shorter episodes. Additionally, patients experienced improvements in gait, more independent mobility, enhanced psychosocial well‐being, and higher quality of sleep, significantly contributing to their overall functionality and quality of life. Specifically, the patient described previously^13^ was never able to walk under caffeine administration, while under theophylline treatment the patient started walking independently. Only minor adverse effects have been reported.

We are aware that there are several aspects of this study that should be considered. First, the retrospective nature of the study introduces a potential bias and limits the ability to draw causal inferences. It is uncertain whether the participants were aware of the results from the initial case report involving the preschool‐aged patient.^13^ Patients #2, #6, and #12 (Table 1) had just started theophylline administration when completing the questionnaire. The relatively small cohort size is attributed to the rarity of ADCY5‐related dyskinesia, which poses challenges to the feasibility of conducting randomized trials.

It has to be considered that the administration of theophylline might not improve sleep quality in ADCY5‐related dyskinesia: Three patients (#1, #6, and #12; Table 1) reported no improvement in sleep quality after theophylline administration. For patient #1, theophylline administration began at an age before the typical symptoms of the disease manifested. As evident from the overall improvement in quality of life for this 2‐year‐old patient, theophylline has a positive effect, but this patient never experienced sleep disruption due to the disease. Patient #6 underwent surgery for DBS prior to theophylline administration, therefore it is difficult to attribute the effects to the theophylline treatment alone. Finally, patient #12 indicated that the reduced sleep quality might also be credited to the concurrent use of diazepam.

In conclusion, while theophylline demonstrates substantial promise as a treatment for ADCY5‐related dyskinesia, further research is warranted to optimize dosing, to better understand long‐term effects, and to explore the benefits of combinational drug therapies, especially regarding the aforementioned limitations.

We are fully aware of the narrow therapeutic window of theophylline, which can, however, be efficiently met by a careful up‐dosing of the drug. The dosage scheme is included in the Data S1. Due to the longstanding widespread use of theophylline in pediatrics in recent decades, there is a large body of clinical experience in the use of the drug, especially in monitoring and avoiding side effects.

Based on our results, we recommend theophylline administration in patients with ADCY5‐related dyskinesia as equivalent treatment option to caffeine. Blood drug levels should optimally range between 15 and 20 mg/L to decrease dyskinetic movements and to improve overall quality of life.

## Author Roles

(1) Research Project: A. Conception, B. Design, C. Execution (informed willing participants, and collected and anonymized the questionnaire responses); (2) Statistical Analysis: A. Design, B. Data Analysis, C. Review and Critique; (3) Manuscript Preparation: A. Writing of the First Draft, B. Review and Critique.

D.T.: 1B, 1C, 2B.

A.M.: 1B, 2B.

A.S.: 1A, 1B, 2A, 3A, 3B.

F.H.: 2B, 2C, 3A.

## Financial Disclosures

A.S. acknowledges financial support from the Deutsche Forschungsgemeinschaft (DFG) (RTG 2467, project number 391498659 “Intrinsically Disordered Proteins‐Molecular Principles, Cellular Functions, and Diseases”, INST 271/404‐1 FUGG, INST 271/405‐1 FUGG), the Federal Ministry for Economic Affairs and Energy (BMWi, ZIM project KK5096401SK0), the region of Saxony‐Anhalt, and the Martin Luther University Halle‐Wittenberg (Center for Structural Mass Spectrometry).

## Supporting information


**Data S1.** Supporting Information.

**Data S2.** Supporting Information.

## Data Availability

The data that support the findings of this study are available from the corresponding author upon reasonable request.
