# Supplementary material for: Treatment Efficacy of Theophylline in ADCY5‐Related Dyskinesia: A Retrospective Case Series Study
Source: Mov Disord. 2025 Mar 13;40(6):1143–7. doi: 10.1002/mds.30170 (PMC12160982; doi:10.1002/mds.30170)
Supplement: Supplementary file 1 — Data S1. Supporting Information. [file MDS-40-1143-s002.pdf]

Dosage of Theophylline (Slow-Release Formulation) for Treating Patients with ADCY5-Related Dyskinesia

Prof. Dr. Andrea Sinz, Institute of Pharmacy, Martin Luther University Halle-Wittenberg, Germany; Email: andrea.sinz@pharmazie.uni-halle.de

Prof. Dr. Andreas Merkenschlager, Department of Neuropediatrics, Hospital for Children and Adolescents, University of Leipzig, Germany; Email: andreas.merkenschlager@medizin.uni-leipzig.de

The use of theophylline as a slow-release formulation offers a specific treatment of patients with ADCY5-related dyskinesia due to its mode of action at the adenosine A_{2A} receptor. Theophylline has been shown to exhibit only minor side effects in children with status asthmaticus that were treated with a low-dose of theophylline (5-7 mg/kg per day). The relatively narrow therapeutic window of theophylline can be addressed by slowly increasing the dose of theophylline and by monitoring theophylline blood levels.

The following dosage scheme has been applied by us:

- A preschool-aged patient with ADCY5-related dyskinesia that had been successfully treated with 150 mg of caffeine (3x 50 mg daily) was treated with 400 mg of slow-release theophylline (2x 200 mg daily).
- Initially, theophylline and caffeine were given in combination, but during the course of the therapy, the daily caffeine dose was reduced over a period of five months until theophylline was administered alone.
- The theophylline dose was gradually increased from ~6 mg/kg/day to ~23 mg/kg/day over a time course of 8 weeks. Theophylline blood levels ranged between 7.5 mg/l to 21.1 mg/l.
- At a theophylline dose of ~12 mg/kg/day (divided into two single doses), the following effects were observed: The patient became more upright, showed increased muscle tone, and was able to stand and walk independently. The quality of sleep improved as dyskinetic movements subsided completely when falling asleep.
- By increasing the theophylline dose to ~23 mg/kg/day, continuous improvements were observed.
- We did not observe any adverse side-effects during theophylline dose titration. Arterial blood pressure, pulse rate, and theophylline-related laboratory parameters were within the given reference ranges.
- The following blood parameters should be regularly checked after administration of theophylline as slow-release formulation:

    Theophylline
    Electrolytes (Na, K)
    Complete blood count
    Creatinine
    Urea
    ASAT
    ALAT
    TSH (hyperthyreosis is possible)
    Blood pressure should also be checked.
